# Detection of Biomolecular Binding Through Enhancement of Localized Surface Plasmon Resonance (LSPR) by Gold Nanoparticles

**DOI:** 10.3390/s90402334

**Published:** 2009-03-30

**Authors:** Hyung Min Kim, Seung Min Jin, Seok Kee Lee, Min-Gon Kim, Yong-Beom Shin

**Affiliations:** 1 Fusion Biotechnology Research Center, KRICT, Daejeon 305-600, Republic of Korea; E-Mails: hyungkim.korea@gmail.com (H-M.K.); smjin@krict.re.kr (S-M.J.); 2 BioMonitoring Research Center, Korea Research Institute of Bioscience and Biotechnology, Yusong-gu, Daejeon, 305–333, Korea; E-Mails: sklee@infopia21.com (S-K.L.); mgkim@kribb.re.kr (M-G.K.)

**Keywords:** Localized surface plasmon resonance (LSPR), gold nano-island, gold nanoparticle, attenuated total reflection (ATR), well chip

## Abstract

To amplify the difference in localized surface plasmon resonance (LSPR) spectra of gold nano-islands due to intermolecular binding events, gold nanoparticles were used. LSPR-based optical biosensors consisting of gold nano-islands were readily made on glass substrates using evaporation and heat treatment. Streptavidin (STA) and biotinylated bovine serum albumin (Bio-BSA) were chosen as the model receptor and the model analyte, respectively, to demonstrate the effectiveness of this detection method. Using this model system, we were able to enhance the sensitivity in monitoring the binding of Bio-BSA to gold nano-island surfaces functionalized with STA through the addition of gold nanoparticle-STA conjugates. In addition, SU-8 well chips with gold nano-island surfaces were fabricated through a conventional UV patterning method and were then utilized for image detection using the attenuated total reflection mode. These results suggest that the gold nano-island well chip may have the potential to be used for multiple and simultaneous detection of various bio-substances.

## 1. Introduction

Impingement of external photons or electrons with specific energies induces collective oscillations of charge densities in metal 3-D nano-structures, which are defined as localized surface plasmon resonance (LSPR). The electromagnetic fields of LSP are confined to the metal nano-structures and hence, LSPR differs from the propagating surface plasmon resonance (PSPR) which is generated at semi-infinitely continuous metal films with the optical systems providing incident photons with the momenta required by the plasmon excitation [1]. LSPR spectra can be easily measured using normal absorption spectroscopy, unlike PSPR. The position and intensity of the LSPR absorption bands are dependent on the dielectric constants near the surfaces of the nano-structures, as well as the sizes, shapes and their composition [2,3]. Therefore, molecular adsorptions on the metal surface will increase the dielectric constant and change the intensities and the wavelengths of the LSPR band. As a result, the adsorptions of chemical or biological molecules onto surfaces of metal nano-structures can be monitored by measuring the absorption spectra of the metal nano-structures.

Label-free detection technologies based on LSPR for biosensing applications have been reported using different types and shapes of metal nano-structures [4–8]. Among the various nano-structures of noble metals, metal nano-islands (NI) can be easily and reproducibly fabricated by conventional evaporation and heat treatment. In addition, the relatively strong adhesion of NI films to a substrate gives the NI sensors mechanical robustness [6]. Recently, we have developed a novel approach for the detection of biomolecules, in which LSPR optical detection with gold NI was implemented to analyze binding of proteins to surfaces functionalized with the corresponding high affinity ligands. This method was used to rapidly detect recombinant GST-tagged hIL6 expressed in *Escherichia coli* by attenuated total reflection (ATR) image measurements [7]. In our previous study, the analyte molecules were directly captured by gold NI surfaces functionalized with small sized receptors such as biotin or glutathione molecules. In this case, we were able to observe a sufficient increase in the LSPR signal when the analyte molecules adsorbed to the gold NI, even at low analyte concentrations. However, when large molecules, such as proteins, are used as the receptors, the sensitivity in detecting binding events with LSPR is expected to be noticeably lower. This is expected since the penetration depth of the LSP field in metal 3-D nanostructures is at most a few tens of nanometers. Actually, this expectation was indirectly demonstrated in a previous investigation, where the absorbance only slightly increased even though a great deal of human serum albumin (HSA) was bound to gold nanoparticles (NP) modified with the anti-HSA on quartz substrates [5].

**Figure 1.**
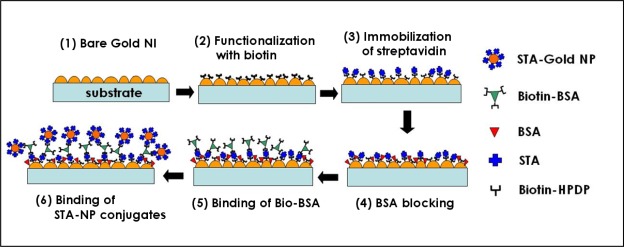
Schematic illustration representing the modification procedure of gold NI surfaces and the bindings of biomolecules.

In this study, we have developed another approach, in which gold NPs were employed to enhance the sensitivity in monitoring the binding of analytes to large receptors such as proteins with LSPR using gold NI chips. Using this approach with gold NP-streptavidin (STA) conjugates (Figure 1), we were able to detect the specific binding of biotinylated BSA (Bio-BSA) to STA immobilized on gold NI surfaces with high sensitivity. In addition, the gold NI chip was also extended to a type of micro-well array and we demonstrated that the well-array chip could be readily utilized as a simple assay tool, with the help of ATR imaging, for detection of various biological species.

## 2. Results and Discussion

### 2.1. Affinity-based bioassay with normal transmission measurements using gold NI chip

**Figure 2.**
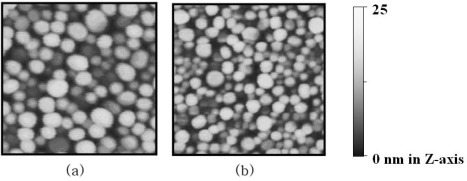
2-D AFM images of (a) gold NI film covered with Bio-BSA molecules and (b) STA-gold NP conjugates subsequently bound to Bio-BSA on gold NI surface. The scanned areas are 500 × 500 nm^2^.

As already reported in previous studies [7,9,10], heat treatment after the formation of gold NI leads to a blue shift of the LSPR band of gold NI to the visible range and consequently the absorption peak appears near 550 nm, similar to the absorption spectra of gold nanoparticles immobilized on transparent substrates. This phenomenon occurs because of the changes in the morphology of gold NI film. An AFM image of the gold NI film after Bio–BSA was conjugated to the STA surface of gold NI chip is shown in Figure 2(a). The average height and diameter of the gold NI were 15.9 nm and 69.2 nm, respectively, which were similar dimensions to those reported in a previous study [7]. After incubation with STA-NP solution, STA-gold NP conjugates, which are smaller than gold Nis, were found to be randomly bound between gold NIs (Figure 2(b)). These results indicate that the STA-NP conjugates were successfully anchored to Bio-BSA bound on gold NI.

**Figure 3.**
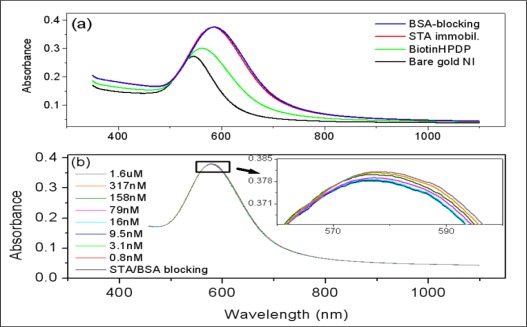
Changes in the absorption spectra of (a) the gold NI film due to the modifications steps which proceed from bare to BSA-blocking and (b) the gold NI films after the incubation of various concentrations of Bio-BSA following BSA-blocking. The peak region of LSPR band was enlarged in the inset plot.

Figure 3(a) shows the absorption spectra of gold NI in the bare state and the consecutively modified states after the subsequent modifications with biotin-HPDP, STA and blocking BSA. As shown in this figure, the intensity of maximum absorbance increased and the wavelength of LSPR band peak (λ^peak^) shifted to the longer wavelengths after each subsequent modification step of the gold NI surfaces. This occurred because as the number of molecules adsorbed on gold NI surfaces increased, there was a corresponding increase in the dielectric constant of the local regions near the interfaces of gold NI. This was especially true when large amounts of STA molecules (1.5 μM) were bound to the biotin surfaces on gold NI films, which also led to the color change of gold NI films from purple to violet, as observed by the naked eye.

Figure 3(b) represents the LSPR spectra of gold NI films incubated with concentrations of Bio-BSA that ranged from 0.8 nM to 1.6 μM. Unlike the large changes in the intensity of LSPR band observed in the successive immobilization procedure shown in Figure 3(a), the increases in the intensities of LSPR bands were so slight that no changes appeared to have occurred as the concentration of bio-BSA increased. However, magnification of the peak region of LSPR bands in the inset plot of Figure 3(b) revealed trivial increases in the intensities of LSPR bands at increasing Bio-BSA concentrations.

**Figure 4.**
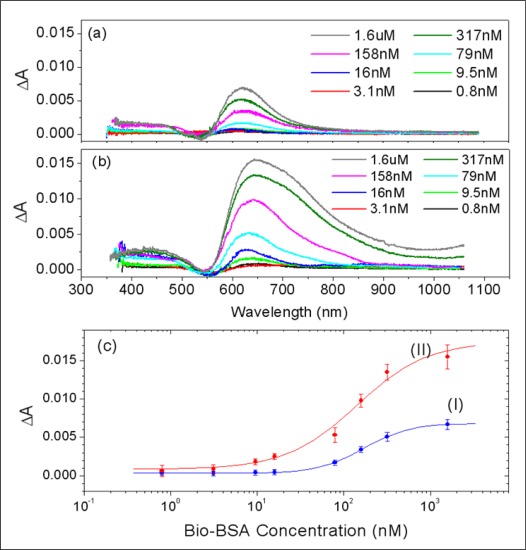
Difference spectra of absorbance for (a) the gold NI chips incubated at various Bio-BSA concentrations (b) the same gold NI chips after subsequent incubations with solutions of STA-gold NP conjugates. (c) Differences in absorbance (I) and (II) at 640 nm in Figure 4(a) and (b), respectively, as a function of Bio-BSA concentration. The error bars show the deviations between six measured values from three different positions on two different gold NI chips.

For a more detailed inspection of the changes in the LSPR bands at the various concentrations of Bio-BSA, difference spectra were obtained by subtracting the absorbance spectrum of the STA-modified gold NI film from those after incubation with the Bio-BSA solutions. As shown in Figure 4(a), the difference spectra of absorbance displayed no meaningful changes in the intensities of LSPR bands up to 16nM of Bio-BSA and that the maximum value around 640nm did not began to increase until a concentration of 79 nM was used. The calibration curve of the absorbance change at 640 nm as a function of Bio-BSA concentration [Plot I in Figure 4(c)] adopted the typical sigmoidal form. This indicates that binding of Bio-BSA to STA on the gold NI chip can be reliably detected only when the concentration of Bio-BSA is higher than 70 nM. This result was entirely different from the finding of a previous study [7], in which binding of STA was reliably observed down to 0.6 nM when a biotin-modified gold NI sensor chips was used. In addition, this result was comparable with another study where gold nanoparticles were used [11].

In our previous study, the bindings of STA and recombinant GST-tagged protein molecules to gold NI surface functionalized with small sized receptors, biotin and glutathione, respectively, resulted in sufficient changes in the intensities of LSPR bands of gold NI. In the present study, however, large molecules such as proteins were used as receptors in lieu of small linkers. Therefore, the difference in size of receptor molecules conjugated to gold NI may explain the discrepancy observed between these results.

The penetration depth of the LSP field to the ambient media in metal 3-D nanostructures is approximately a few tens of nanometers [12], which is about one tenth shorter than a PSP field created in 2-D metal nano-films using a prism coupler [13–16]. In other words, the amplitude of LSP field is attenuated at an extremely rapid rate in the direction normal to the surface of metal. As a result, analyte molecules that were adsorbed to large receptors conjugated to NI surfaces would be very restrictively exposed to the LSP field. Therefore, one would expect that the sensitivity in detecting intermolecular binding events when using metal NI with large receptors would be conspicuously lower than when using small receptors. In fact, this result is consistent with a previous investigation [5], in which the absorbance increased by only a slight degree even though a great deal of HSA was bound to gold NPs modified with the anti-HSA (∼ 150 kDa) on quartz substrates.

To enhance the sensitivity in biomolecular sensing using gold NI films, we used the concept of the sandwich assay and made use of STA-gold NP conjugates to utilize metal nanoparticles with a large dielectric constant as the labeling material.

Figure 4(b) presents the difference spectra of the absorbance obtained by subtracting the absorbance spectrum of the STA-modified gold NI film before interaction with Bio-BSA from those of gold NI films covered with the various concentrations of Bio-BSA molecules as described in Figure 4(a) after incubation with solutions of STA-gold NP conjugates. The intensities of the difference spectra, in this case, increased to a larger extent than before, thus STA-gold NP conjugates enhanced the difference in LSPR intensities in a Bio-BSA concentration dependant manner [Figure 4(b)]. Especially, it should be noted that the increase in absorbance near 700 nm is rather conspicuous. This originates from the plasmon-coupling effect between Au NI and NP, which leads that their LSP band shifted to much longer wavelength region than their original positions in the range of 520 ∼ 560 nm. Consequently, the intensity of the spectrum at even 9.5 nM of Bio-BSA showed a significant enough increase in the absorbance difference to confirm binding of Bio-BSA with STA on gold NI surface.

The calibration curve of the absorbance change at the peak in Figure 4(b) also displayed the characteristic sigmoidal function of Bio-BSA concentration [Plot II in Figure 4(c)]. This curve indicated that the specific adsorptions of Bio-BSA onto gold NI surfaces functionalized with STA can be monitored down to about 6 ∼ 7nM of Bio-BSA when STA-gold NP conjugates are used, which is 11-times lower than the limit of detection (LOD) when using label-free methods.

### 2.1. ATR image measurement for the detection of multiple species using gold NI well chip

We have shown in a previous report [7] that the absorbance intensity of gold NI film measured by the ATR mode is considerably larger than that measured by the normal transmission mode; hence, we expect that even minute differences in the absorption intensity of each sample could be significantly amplified, such that they would be detectable when measured in the ATR mode with a prism. Accordingly, we also tried to implement multiple detection based on ATR imaging with gold NI well chips to detect binding of Bio-BSA with STA on sensor chips.

**Figure 5.**
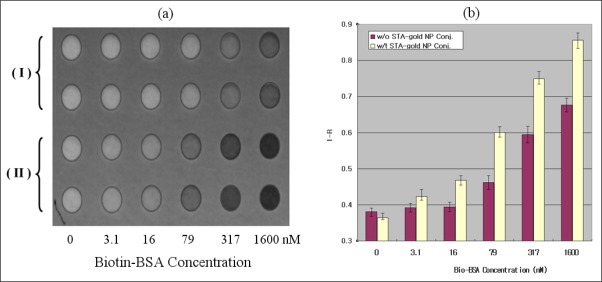
(a) ATR image of gold NI chip including several well surfaces incubated with (I) only various concentrations of Bio-BSA and (II) the solution of STA-gold NP conjugates after the incubation with Bio-BSA. The experiments were duplicated. (b) Plot of the values of (*1-Ri*) for each well. The error bars show the deviations between the six measured values from the duplicated wells of for the identical concentration of Bio-BSA on three different chips.

Figure 5(a) shows an ATR image at 647 nm of the SU-8 wells chip of gold NI surfaces. It should be noted that a larger absorption at the monitored wavelength gives a darker image because the intensity of light decreases when internally reflected at an interface that includes an absorbing media. Therefore, we expect from the normal transmission measurement of Figure 4 that binding of larger amounts of Bio-BSA molecules to STA on gold NI chips lead to the darker ATR images of the micro well surface. In accordance with our prediction, it was not difficult to distinguish the differences in the ATR images of gold NI wells incubated with different concentrations of Bio-BSA solutions even by the naked eye.

For quantitative analysis of the light intensity of the well image, the pixel values in the central 70% region of each well were averaged out to the value *I_i_*. A well with a bare glass surface was used as a reference and the averaged pixel-value of the reference well, *I_0_*, was used to normalize the reflectivity (*I_i_/I_0_* = *R*_i_) where *i* is introduced to distinguish each well.

The 2-D intensity profile of the incident beam used in this study exhibited a broad Gaussian distribution; hence, it is likely that each well had a different *R* value even though the wells were identical. To eliminate this error, the normalized reflectivity of each well was corrected by using the values of the background pixels in the SU-8 region near the boundary of each well. Finally, the values of (*1−R_i_*) from each well with the concentration of Bio-BSA were plotted in Figure 5(b). These results indicate that detection with STA-gold NP conjugates was much more sensitive than without them in ATR image measurement as well as in the normal transmission mode. Consequently, 3.1 nM of Bio-BSA in the PBS solution could be indirectly detected using STA-gold NP conjugates.

These results suggest the intriguing possibility that the detection of binding through ATR imaging in combination with gold NI well chips using gold NP conjugates could be useful for multiple sensing of different biomolecules.

## 3. Experimental Section

### 3.1. Formation of gold nano-island(NI) films on glass substrates

The formation of Au NI on glass substrates was described previously [7]. In brief, clean glass substrates were treated with 3-mercaptopropyl trimethoxysilane instead of transition metals to increase the adhesive strength between the gold film and the glass surfaces without damping the LSPR in gold NIs. The modified glass chips were coated with gold films using an electron beam evaporator. The gold films were deposited on the glasses at an average deposition rate of ∼0.05 Å/s under ∼1×10^−6^ torr and the final thickness of gold film was 65 Å, which is near to the optimal thickness for the formation of nano-island exhibiting a clear LSP peak [9,10]. The glass chips coated with gold films were heat-treated at 210 ^°^C for 60 h in a N_2_-atmospheric furnace. The gold NI chips were then treated with a solution containing a 1:1 volume ratio of CH_2_Cl_2_ and ethanol to stabilize against the blue shift of the plasmon band, which typically appears in metal nano-islands immersed in solvents [3,9].

### 3.2. Modifications of gold NI surfaces and bindings of Bio-BSA with gold NI surfaces

The gold NI surfaces was modified with biotin as follows; a self assembled monolayer (SAM) of *N*-[6-(biotinamido)hexyl]-3′-(2′-pyridyldithio)propionamide (Biotin-HPDP: Pierce, USA) was formed on gold NI surfaces by incubating in 0.1 mM biotin-HPDP ethanol solution for 4 h at room temperature and then thoroughly rinsing and drying the gold NI chips. Next, diluted solutions of streptavidin (100 μg/mL) from *Streptomyces avidinii* (1 mg/mL STA: Sigma-Aldrich, USA) were directly loaded on the biotin-functionalized gold NI chips and incubated for 1 h at 25 ^°^C in a Petri-dish hermetically sealed to prevent the solution from evaporating. This was followed by rinsing five times with PBS. STA-modified gold NI chips were incubated for 3 h in a 1 mg/mL bovine serum albumin (BSA) solution to minimize nonspecific bindings.

Bio-BSA (2 mg/mL biotinylated Bovine Serum Albumin: ThermoFisher, USA) was diluted to various concentrations in PBS. The diluted solutions (100 μL) of Bio-BSA were deposited onto the STA-functionalized gold NI chips and then the chips were incubated at 25^°^C for 1 h in a Petri-dish hermetically sealed. Subsequently, the gold NI chips were rinsed five times with PBS and dried under a N_2_ stream.

### 3.3. Synthesis of STA-gold NP conjugates for enhancement of the LSPR signals

The pH of 1 mL of colloidal gold (20 nm diameter, BB International, UK) with an optical density of 1.0 (7 × 10^11^ particles) at 520 nm was adjusted to 8.5 with 100 μL of 0.1 M borate buffer (pH 8.5). Gold NPs were conjugated with STA by incubating 100 μL from 1 mg/mL stock STA solution for 1 h and then blocked with 1% BSA for 30 min at room temperature. The STA-gold NP conjugates were washed four times with PBS containing 0.1% BSA by centrifugation at 10^4^ G for 20 min. at 4°C. The STA-gold NP conjugates were stabilized with 0.1% BSA and 0.05% sodium azide. The conjugate solution was re-suspended into the same buffer solution to a final optical density at 520 nm of 1.8 ∼ 2.0. The gold NI chips previously reacted with the various concentrations of Bio-BSA solutions were immersed in the solutions of STA-gold NP conjugates for 1 h. The incubated chips were carefully rinsed with PBS and DI water, followed by drying under a weak stream of N_2_.

### 3.4. Measurements of LSP bands and surface morphologies

The changes in LSPR spectra of gold NIs resulting from the binding of Bio-BSA molecules and the STA-gold NP conjugates on STA-modified NI chips were observed under the atmospheric condition using a DU 800 spectrophotometer (Beckman-Coulter, USA) with optical resolution of 0.5 nm. Surface morphologies of gold NI films before and after binding of STA-NP conjugates were imaged with an atomic force microscope (AFM: Digital Instruments, USA) in non-contact mode.

### 3.5. Fabrication of gold NI well chip and measurements of affinity bindings to the gold NI well chips using the ATR mode

The fabrication procedure for the gold NI well chips was described previously [7]. In brief, 10 μm-thick layers of SU-85 were formed on a non-heated gold NI chip by spin coating and then, preliminary baked on a hot plate. A 6 × 4 array of ϕ800 μm-wells was patterned on each NI chip by UV lithography. Subsequently, the gold NI chips patterned with SU-8 wells were heat-treated at 210 ^°^C for 60 h in a N_2_-atmosphere furnace.

The functionalization of the annealed gold NI well chips with STA was carried out using the same procedure as described previously (*vide supra*): 0.5 μL of Bio-BSA solutions with various concentrations were deposited into the wells on the chips and the well chips were incubated for 1 h at 25 ^°^C in a hermetically sealed Petri-dish. The incubated chips were extensively rinsed with PBS. To amplify the changes in the LSPR band of the gold NI due to the binding of Bio-BSA, solutions (0.5 μL) of STA-gold conjugates were deposited onto each well and then the well chips were incubated and washed in the same manner as above, followed by drying with mild stream of N_2_.

The ATR imaging set-up for LSPR detection was discussed previously [7]. Briefly, a white light from a quartz tungsten-halogen (QTH) lamp was collimated by a lens and a pinhole, and then passed through a narrow bandpass filter (λ_0_ = 647 nm, FWHM = 1 nm). The gold NI well chip was optically matched with a BK7 prism coupler using index matching oil. The ATR images from the gold NI well chips were focused to a CCD camera by a combination of lens. The images were then stored and analyzed digitally by a commercially available program.

## 4. Conclusions

The gold NI film chips prepared by evaporation and heat treatment were employed as optical sensors on the basis of LSPR to detect binding of Bio-BSA molecules to the gold NI modified with STA. From these experiments, we were able to reliably detect binding of Bio-BSA at only concentrations higher than 79 nM using the label-free method. In contrast, when the sandwich method using STA-gold NP conjugates was employed we were able to observe Bio-BSA – STA-modified gold NI surface binding at 7 nM and 3.1 nM Bio-BSA in the normal transmission and ATR image mode, respectively. These results show that sensing via the combinations of gold NI chip and gold NP conjugates is 11-times more sensitive than the detection using label-free method without gold NP conjugates. In addition, our results suggest that gold NI well chip accompanied by gold NP conjugates using detection through ATR imaging may be a simple and efficient biosensing tool for multiple detection of different biomolecules.
